# Epidemiology of streptomycin resistant *Salmonella* from humans and animals in Ethiopia: A systematic review and meta-analysis

**DOI:** 10.1371/journal.pone.0244057

**Published:** 2020-12-17

**Authors:** Getachew Mengistu, Getiye Dejenu, Cheru Tesema, Balew Arega, Tadesse Awoke, Kassahun Alemu, Feleke Moges

**Affiliations:** 1 Medical Laboratory Science, College of Health Sciences, DebreMarkos University, Debre Marqos, Ethiopia; 2 Department of Medical Microbiology, School of Laboratory and Biomedical Science, College of Medicine and Health Science, University of Gondar, Gondar, Ethiopia; 3 Department of Public Health, College of Health Sciences, DebreMarkos University, Debre Marqos, Ethiopia; 4 Yekatit 12 Hospital Medical College, Addis Ababa, Ethiopia; 5 Department of Epidemiology and Biostatistics, Institute of Public Health, College of Medicine and Health Science, University of Gondar, Gondar, Ethiopia; University of Lincoln, UNITED KINGDOM

## Abstract

**Background:**

Streptomycin is used as an epidemiological marker in monitoring programs for antimicrobial resistance in *Salmonella* serovars and indicates the presence of pentaresistance. However, comprehensive data on streptomycin resistant *Salmonella* among human, animal, and animal products is lacking in Ethiopia. In this review, we aimed to assess heterogeneity and pooled proportion of *Salmonella serovars* to streptomycin resistance among human, animal and animal products in Ethiopia.

**Methods:**

We conducted a systematic review and meta-analysis of published literature from Ethiopia. We used the MEDLINE/ PubMed, Embase, Cochrane Library, and Google Scholar databases to identify genetic and phenotypic data on *Salmonella* isolates. To determine the heterogeneity and pooled proportion, we used metaprop commands and the random-effects model. Relative and cumulative frequencies were calculated to describe the overall preponderance of streptomycin resistance isolates after arcsine-transformed data. Metan funnel and meta-bias using a begg test were performed to check for publication bias.

**Results:**

Overall, we included 1475 *Salmonella* serovars in this meta-analysis. The pooled proportion of streptomycin resistance was 47% (95% CI: 35–60%). Sub-group analysis by target population showed that the proportion of streptomycin resistance in *Salmonella* serovars was 54% (95% CI: 35–73%) in animal, 44% (95% Cl: 33–59%) in humans and 39% (95% CI: 24–55%) in animals products. The streptomycin resistant *Salmonella* serovars were statistically increasing from 0.35(95% CI: 0.12–0.58) in 2003 to 0.77(95% CI: 0.64–0.89) in 2018. The level of multidrug-resistant (MDR) *Salmonella* serovars was 50.1% in the meta-analysis.

**Conclusion:**

We found a high level of streptomycin resistance, including multidrug, *Salmonella* serovars among human, animals, and animal products. This resistance was significantly increasing in the last three decades (1985–2018). The resistance to streptomycin among *Salmonella* serovars isolated from animals was higher than humans. This mandates the continuous monitoring of streptomycin use and practicing one health approach to preventing further development of resistance in Ethiopia.

**Registration:**

We conducted a systematic review and meta-analysis after registration of the protocol in PROSPERO (**CRD42019135116**) following the MOOSE (Meta-Analysis of Observational Studies in Epidemiology).

## Background

*Salmonella* is a major public health problem affecting both humans and animals. Most mammals and birds can be reservoirs for *Salmonella* as well as many other vertebrates. Some plants may also be able to act as reservoirs [1, 2]. This bacterial species cause gastroenteritis, typhoid fever, non-typhoidal *Salmonella*, miscarriage, and bacteremia, depending on the types of serovars and the hosts [3, 4]. The infection is transmitted through unhygienic living conditions, sharing of houses between animals and humans, and consumption of raw or undercooked animal-origin food items [5]. The problem is much worse in developing countries where the aforementioned means of transmission are common [5].

The emergence and spread of drug-resistant *Salmonella* serovars are one of a current global concern [6]. The widespread use of antimicrobials at suboptimal doses and their prophylactic use in livestock, companion animals, and humans led to the emergence of drug-resistant *Salmonella* serovars to different groups of antimicrobial agents [7]. The interaction of humans and animals though the food chain is crucial in the transmission of these resistant bacteria [8]. This is multidimensional nature of the problems is currently recognized, and a comprehensive one health research agenda is proposed to address antimicrobial resistance(AMR), mainly among foodborne pathogens such as *Salmonella* for aminoglycosides, quinolones, cephalosporins and other related antimicrobials [9].

Streptomycin is an aminoglycoside antimicrobial and was one of the first antimicrobial agents to be discovered in the early 1940s [10]. The drug has been used for the treatment of animal and human *Salmonella* infections since its discovery [10]. The resistance level of *Salmonella* serovars is increasing globally for this antibiotic. The most common enzymes associated with this antibiotic resistance are acetyltransferases, phosphotransferases, and nucleotidyltransferases, which modify and inactivate streptomycin and other aminoglycosides [9]. The *Salmonella* serovars (*Salmonella enterica)* resistant to Ampicillin, Chloramphenicol, and other related antibiotics [11] are also use these enzymes. Therefore, detection of streptomycin resistance is potentially used as an epidemiological marker in monitoring AMR programs among *Salmonella* serovars.

Ethiopia is particularly at risk of *Salmonella* infection, including the drug resistance serovars, as 80% of households have direct contact with domestic animals, creating a chance for infection and spread of disease [12]. For example, a study in the country revealed that there is a transmission of drug-resistant *Salmonella* among humans and animals [13]. The study also showed resistant to first and second-line drugs used in the therapeutic management of salmonellosis, including streptomycin resistance [13]. Despite the medical and veterinary significance of the disease, surveillance and monitoring systems are not in place and the pharmaco-epidemiology of streptomycin resistant *Salmonella* serovars is not adequately described in Ethiopia. Therefore, this review aimed to answer the following questions: 1) what is the pooled proportion of streptomycin resistant *Salmonella* serovars among the studies included in this review from Ethiopia? 2) Is the pooled proportion of streptomycin resistant *Salmonella* serovars different among animals, humans’, and animal products in Ethiopia?

## Methods

### Study design and data sources

We performed a systematic review and meta-analysis of published studies in Ethiopia. Before the data extraction, the protocol was registered in PROSPERO (International prospective register of systematic reviews) (CRD42019135116), following the MOOSE (Meta-Analysis of Observational Studies in Epidemiology) guideline. For this, we searched original studies from MEDLINE/PubMed, Google Scholar, Cochrane Library, and African Journals Online (AJOL) databases. We used Preferred Reporting Items for Systematic Reviews and Meta-Analyses (PRISMA) [14, 15] to report the study (S1 Checklist). The electronic search was performed to avoid duplication using the following keywords: streptomycin-resistant *Salmonella* AND Ethiopia AND (‘systematic review’ OR ‘meta-analysis'). Multiple search strings (medical subject headings [MeSH], title/abstract [TIAB]] and text words [TW]) were tried to identify primary studies. The search string that enabled the location of most of the studies was the following: streptomycin AND *Salmonella* AND Human OR animal AND Ethiopia. In addition, we made a search for cross-referencing of the identified original articles. We did the last search on April 11, 2019.

### The study selection

In the first step, all eligible articles which address the study objectives were retrieved by reviewing their title and abstract. In the second step, we deeply assessed every article against the inclusion criteria. The inclusion criteria were:—(i) laboratory-based study reporting streptomycin resistance of *Salmonella* either from humans, animals, or animal products (ii) published in English, (iii) studies conducted in Ethiopia. We excluded letters, communications, and reports with unclear data and those studies which did not include streptomycin antibiotic susceptibility result. We also did not consider studies reiterated findings from the already included studies.

### Data extraction

With the help of a standardized data abstraction format prepared in Microsoft Excel, we (GM and GD) extracted the following characteristics of the study:-first author, year of publication, year of study, region, target population (humans, domestic animals, and animal products), and sampling population (health-care/nosocomial, community, food handler’s, animal species/products, animal handlers, and health status of the sample population), sample size, sampling method, sample type, drug susceptibility test (DST),numbers of isolates, numbers of isolates subjected to DST, numbers of MDR strains, number of streptomycin resistant serovars, and minimum inhibitory concentrations (MICs)/zone diameters (ZD).

### Quality of studies and risk of bias

We use Joanna Briggs Institute (JBI) critical appraisal tool to assess the methodological quality of each study. This tool was used to detect the occurrence of any real evidence of bias based on (i) target population (humans vs. animals vs. animal products), (ii) sampling population (humans–health-care/nosocomial, community, food handler’s; animals/animal products, abattoir, dairy farms, poultry farm), (iii) sample size (n≤384 vs. higher; n = Z^2^PQ/d^2^; where Pexp = 0.5), (iv) sampling method (probability vs. non-probability). In each sampling population, studies with the following characteristics were considered as low-risk studies: sample size (>384) and prospective/consecutive sampling [16]. The Begg and Mazmudar rank correlation test was used to assess bias across studies (small study effects) [17].

### Data analysis

We use Microsoft office excel 2013, Stata (Version 15.1) and EpiInfoTM (version 7.2) for data extraction, entry, and data analysis. Then, we presented a detailed description of the original studies in a table and forest plot. The level of streptomycin resistance was classified as: rare (<0.1%), very low (0.1% -1%), low (>1–10%), moderate (10% -20%), high (20% - 50%); very high (50% - 70%) and extremely high, (>70%) [18].

We determined the pooled estimate of the proportion of streptomycin resistant *Salmonella* using the DerSimonian-Laird for random-effects meta-analysis (random-effects model). We summarized the drug resistance level of *Salmonella* for streptomycin in each study by calculating the proportion in a 95% confidence interval. To normalize data distribution, Arcsine transformation was used and the result was presented using the original probability scale after corresponding back transformation [19].

We tested the possible cause of publication bias and heterogeneity across studies by means of the Cochrane Q test (presence of heterogeneity) and I^2^ statistics (amount of heterogeneity) [20]. The existence of heterogeneity was verified using the Cochrane Q test (P < 0.10 indicates statistically significant heterogeneity). The I^2^ test to measure the level of heterogeneity between studies with the values of 25% (low heterogeneity), 50% (medium heterogeneity), and 75% (high heterogeneity) was used. The Begg’s rank correlation test and Egger weighted regression test was used to statistically assess publication bias. A p-value of< 0.05 was considered indicative of statistically significant publication bias [17].

We performed a sensitivity test to identify which article is the main determinant of the pooled result, and the primary cause of heterogeneity. The test excludes each study one by one in the analysis to show the change in pooled effect size and its heterogeneity. If the point estimate of pooled effect after excluding a study lies within the 95% CI of the overall pooled effect for all studies as a whole, we assumed the study has a non-important impact on the overall pooled effect [17].

We planned subgroup analysis during the study design to examine the source of heterogeneity based on regions, target population and the change in streptomycin resistant *Salmonella* serovars over time.

## Result

### Search and selection of studies

**Fig 1:**
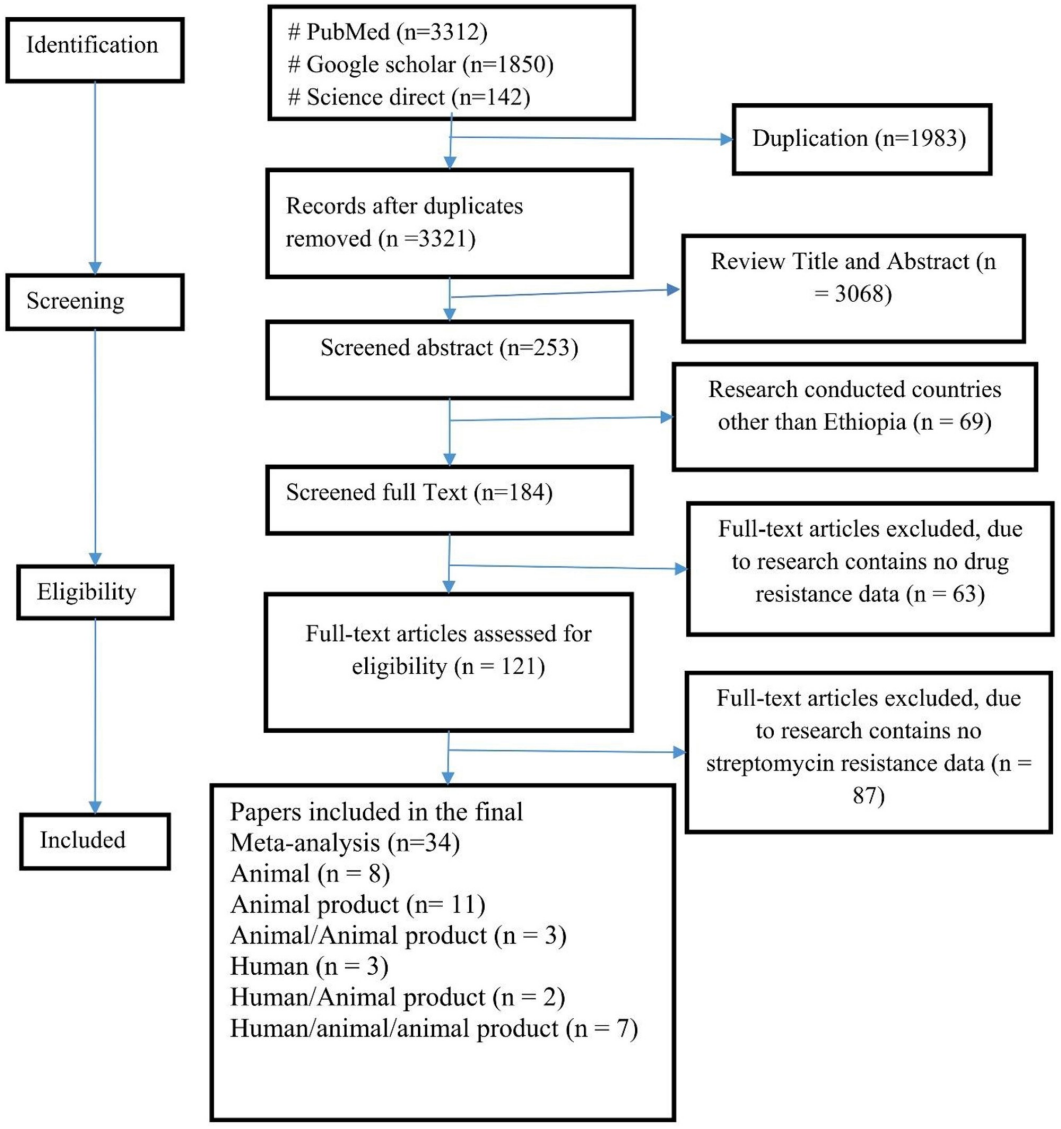
Flowchart of literature search and inclusion/exclusion process.

Overall, we identified a total of 3312 articles using the keywords. Out of these, 3278 were excluded either due to duplication, research conducted outside Ethiopia, or not including the research questions. Others were excluded because of either only reporting *Salmonella* prevalence or resistance for antimicrobial agents other than streptomycin. Lastly, we included only 34 articles in this review and meta-analysis [21–54](Fig 1) (S1 Supplement).

### Characteristics of included studies

Except one retrospective study [21], all others are laboratory-based prospective cross-sectional studies. Based on the source of the specimen for the studies, eleven were conducted among animal products, eight among animal specimens and three used human specimens only. Moreover, seven studies included human, animal, and animal product specimens, three studies included both animal and animal products and the rest three included both human and animal products Table 1.

**Table 1:** Characteristics of included studies by target population and *Salmonella* isolate.

Target population*	Number of Isolates	No of MDR isolates	% of MDR
**Animal**	331	186	56.19
**Animal Product**	430	275	63.95
**Animal/Animal product**	84	58	69.05
**Human**	196	121	61.73
**Human/Animal product**	185	25	13.51
**Human/Animal/Animal product**	249	74	29.72
**Total**	1475	739	50.1

* Some article did not differentiate the bacterial isolates by the target population.

**Fig 2:**
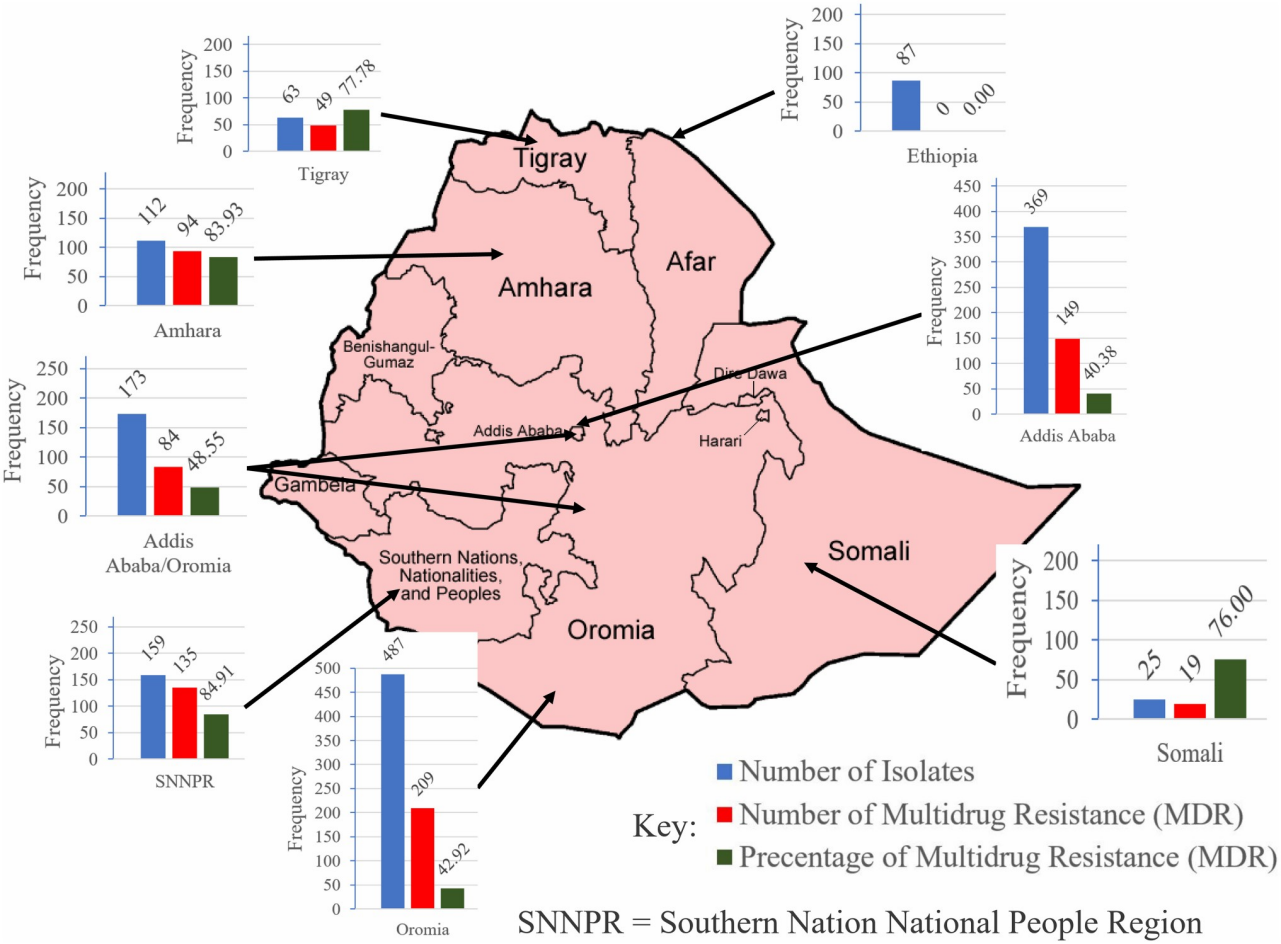
Characteristics of included studies by place of the studies and *Salmonella* isolate.

A total of 1475 *Salmonella* serovars were isolated from 34 included studies. Of these, 430 were only from animal products [22–24, 26, 32, 36, 37, 40, 47, 48, 53], 331 were only from animal specimens [25, 27, 30, 43, 45, 46, 51, 52], 249 were from human/animal/animal product [28, 29, 34, 35, 41, 49, 54] and the rest were from others samples [21, 31, 33, 38, 39, 42, 44, 50] as presented on Table 1. Based on the region, the majority of the isolates (n = 487) were from Oromia region followed by Addis Ababa (n = 369), SNNRP (N = 159) and Amhara (n = 173) [26, 50, 52] (Fig 2).

Antibiotic susceptibility tests were performed for streptomycin in 34 studies and 97.0% (33/34) of them reported that there is at least one resistant serovar among the isolates. Overall, about 50.1% (739/1475) of the *Salmonella* isolates in the included studies were MDR. The majority of the *Salmonella* serovars isolated from animal and animal product specimens were, 69.05% (58/84) MDR, followed by animal products, 63.95(275/430), humans 61.73% (121/196) and animals 56.19% (186/331) Table 1. The proportion of MDR isolates were different across the regions with higher in SNNPR (84.91%, n = 159) followed by Amhara (83.93%, n = 112) and Somali region (77.78%, n = 63).

### Quality assessment and risk appraisal

**Fig 3:**
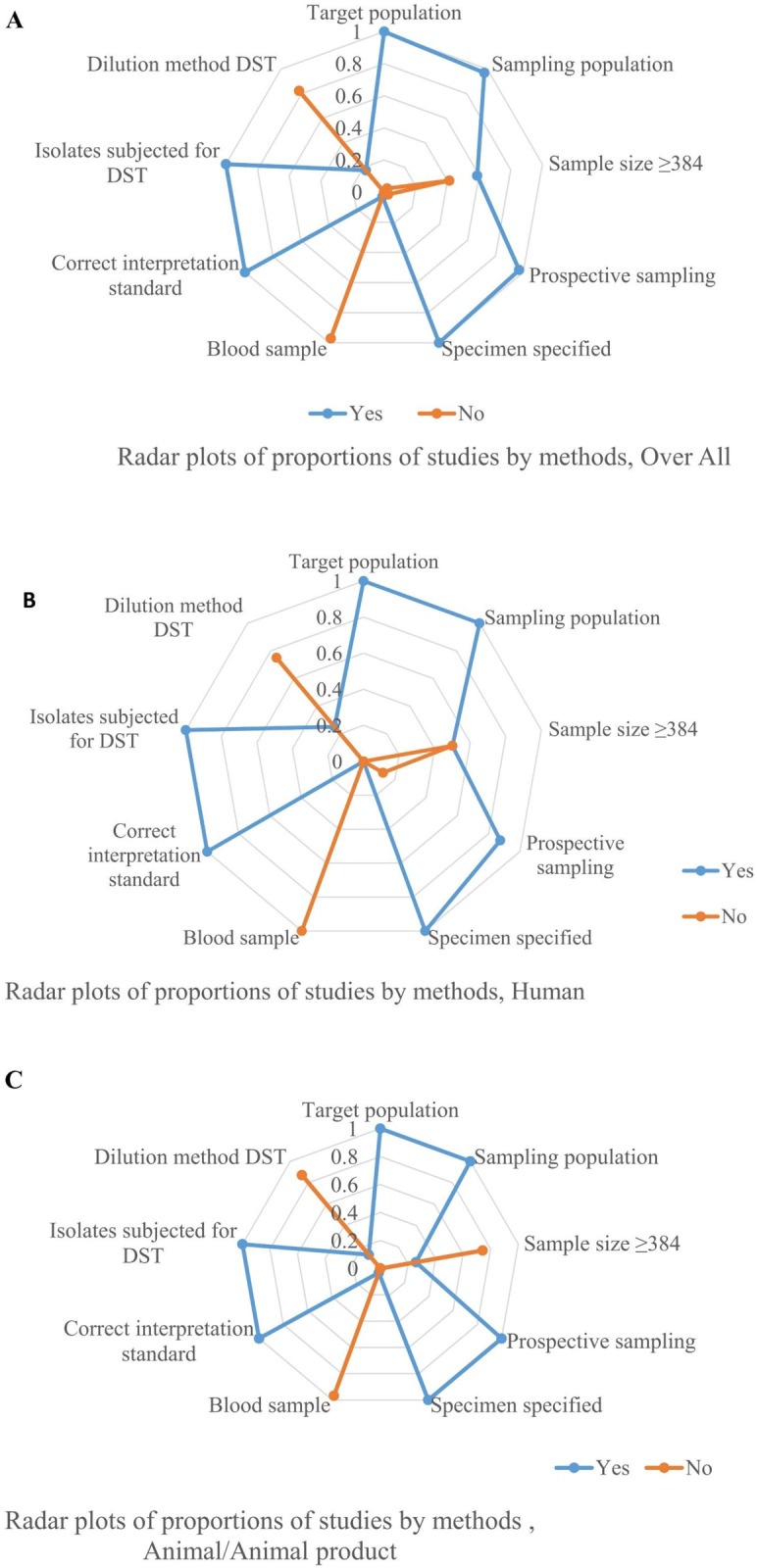
Radar plots of proportions of studies by methods. All studies (A), All human studies (B), All Animal and animal product studies (C).

The methodological qualities and risks of bias of included studies are given in (S2 Supplement). Fig 3 presents the proportion of studies by methods. Target population, sampling population, prospective/consecutive sampling, and specimen specified; correct interpretation and isolates subjected to DST were each reported in 50% or more of the studies (Fig 3A).

Of the studies on isolates of human origins, 90% were relatively homogeneous concerning the target population, sampling population and methods (prospective sampling, specifying the specimen for isolation of the bacteria, correct interpretation of test method, and subjecting all isolates for drug susceptibility testing) Fig 3B. Similarly, the studies on animals and animal products were more than 90% homogeneous with respect to the target population, sampling population, perspective sampling, specimen specified for identification, correct interpretation, and subjecting all isolates to DST Fig 3C. Accordingly, meta- and frequency analyses were performed depending on the number of studies by category, the number of the target population, and risks of bias/heterogeneity.

### Meta analyses

#### Heterogeneity and publication bias

**Fig 4:**
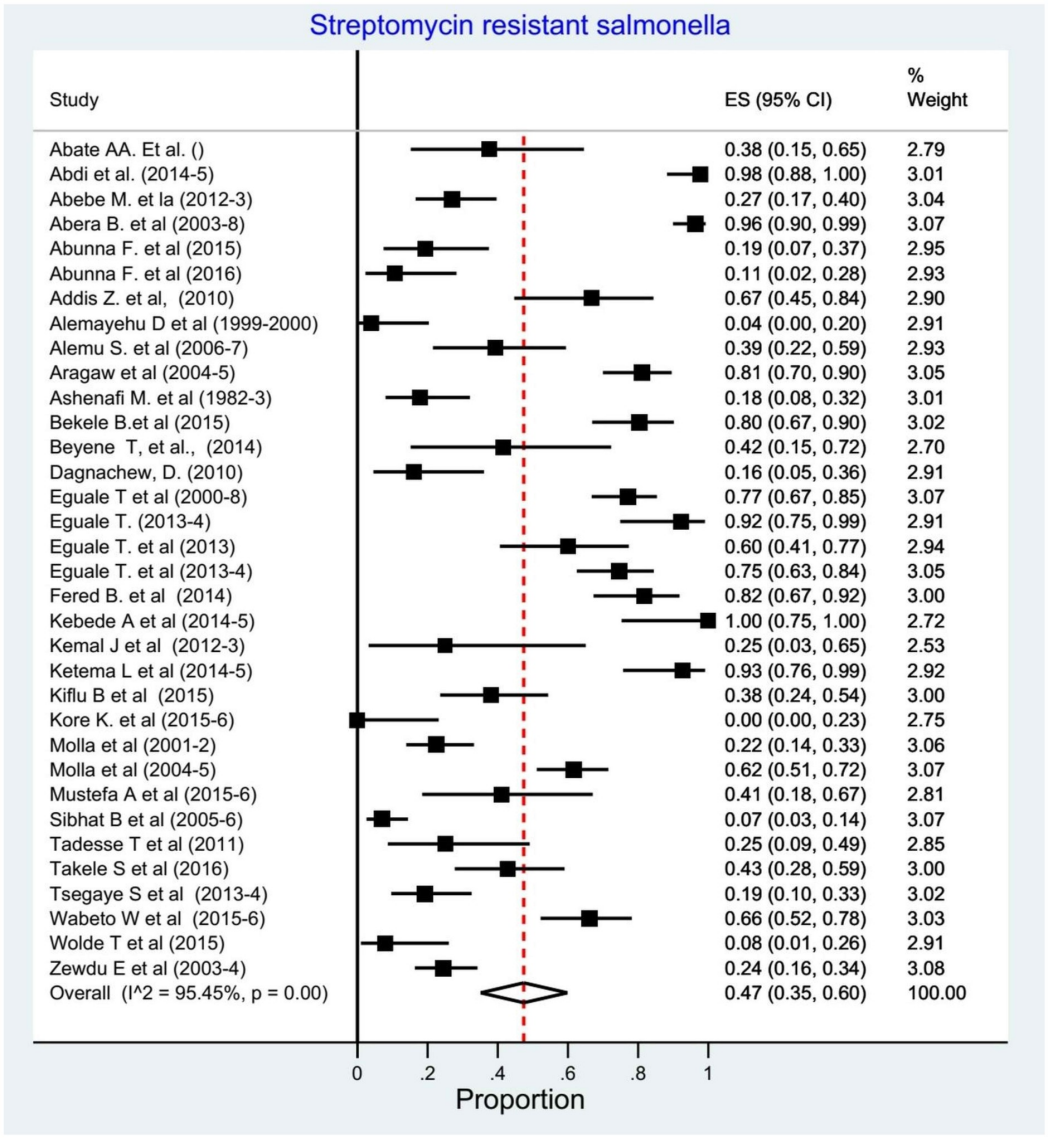
Forest plots of streptomycin resistant *Salmonella* serovars proportions.

**Fig 5:**
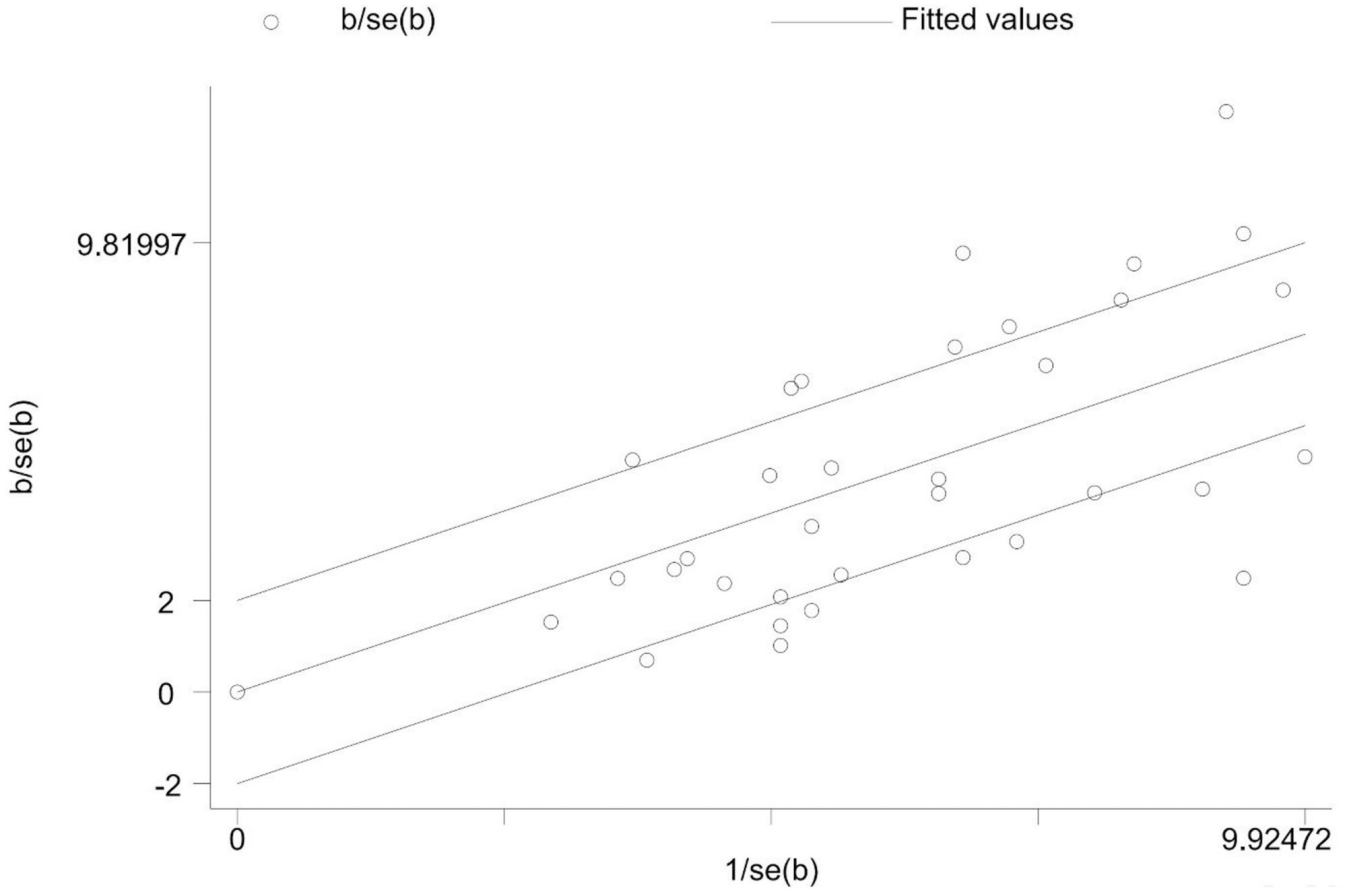
Galbraith plot of streptomycin resistant *Salmonella* serovars proportions among studies.

**Fig 6:**
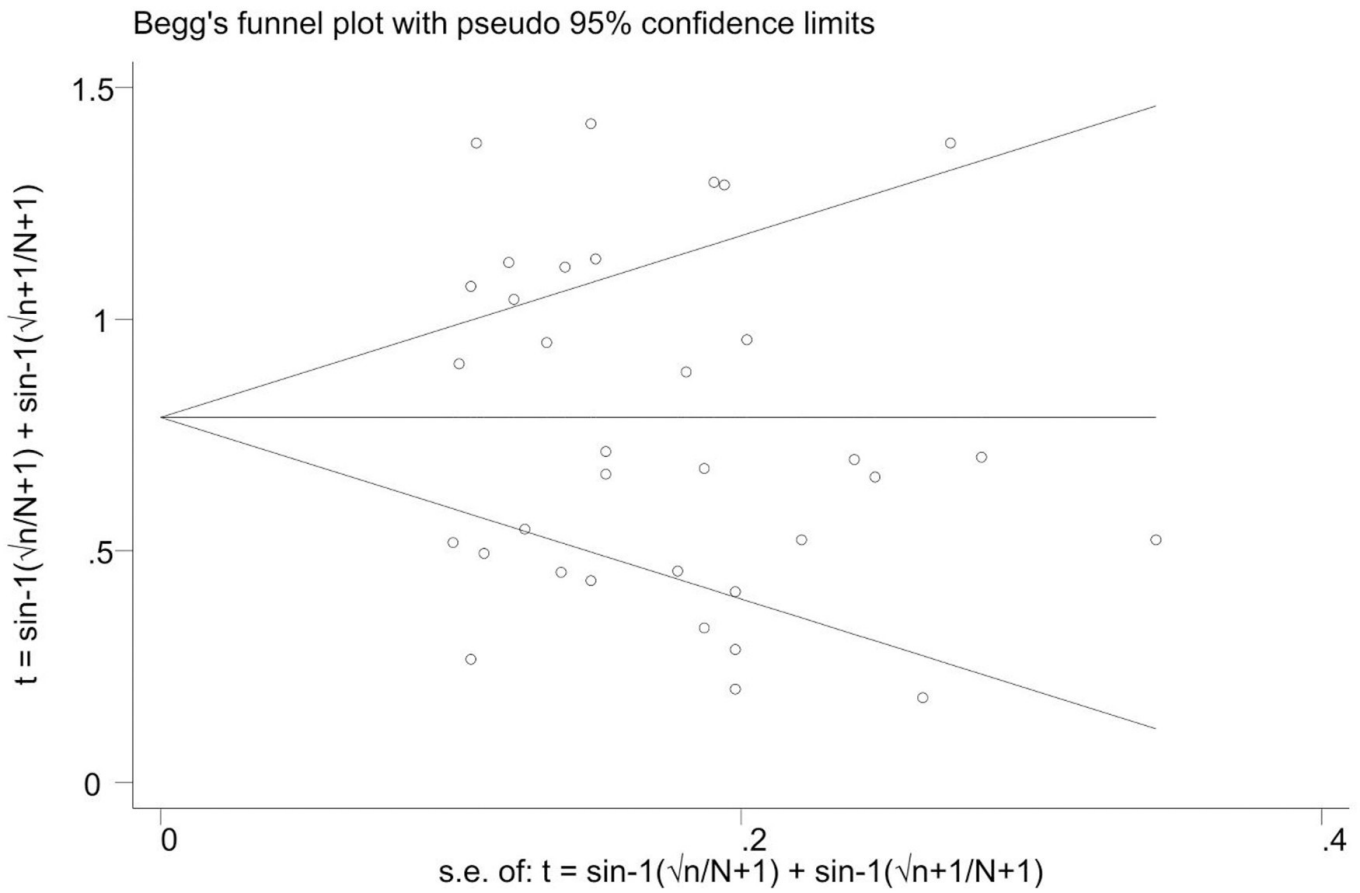
Publication bias (meta-bias) for the proportion of streptomycin resistant *Salmonella* serovars.

**Fig 7:**
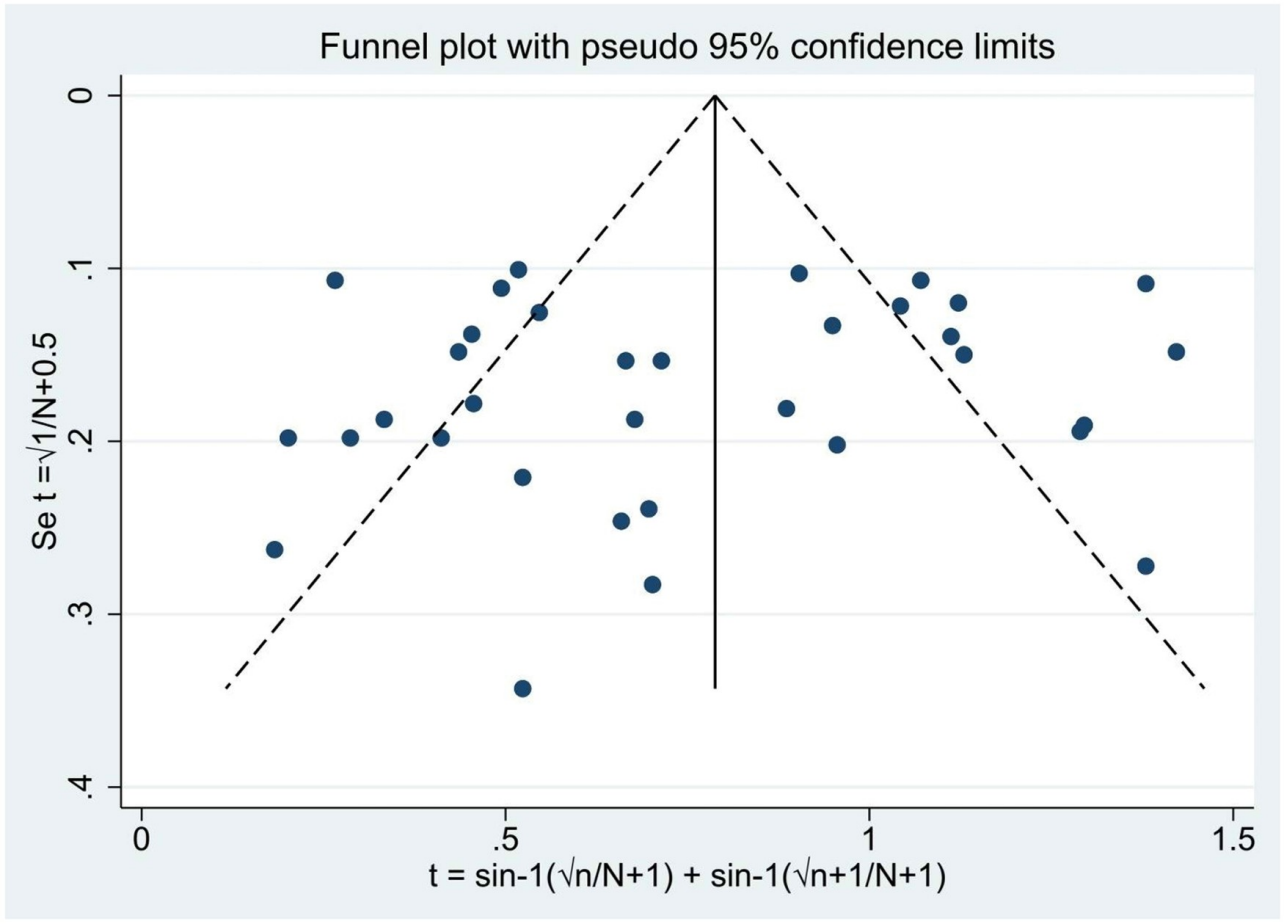
Meta-funnel for the proportion of streptomycin resistant *Salmonella* serovars.

We assessed for heterogeneity and publication bias of studies. The analysis showed substantial heterogeneity among studies (Cochran’s Q test = 725.94, p<0.001; I^2^ = 95.45.0%, p<0.001) (Fig 4). This considerable level of heterogeneity among studies was also visualized by using Galbraith plot (Fig 5) test. The Begg and Mazmudar rank correlation test did not suggest study bias/low study effects (Kendall’s score = -28; P = 0.689 (Fig 6). Meta-Funnel plot (Fig 7) test also showed that there is no publication bias. So, trim and fill analysis is not necessary because there is no publication bias.

#### Prevalence of streptomycin resistant *Salmonella* serovars

We determined the pooled proportion of streptomycin resistant *Salmonella* serovars among 1475 isolates of the 34 studies. The overall pooled proportion of any streptomycin resistant *Salmonella* detection using the random effect model was 0.47% (95% CI: 0.35–0.60, I^2^ = 95.45%, p<0.001) (Fig 4).

#### Subgroup analysis

**Fig 8:**
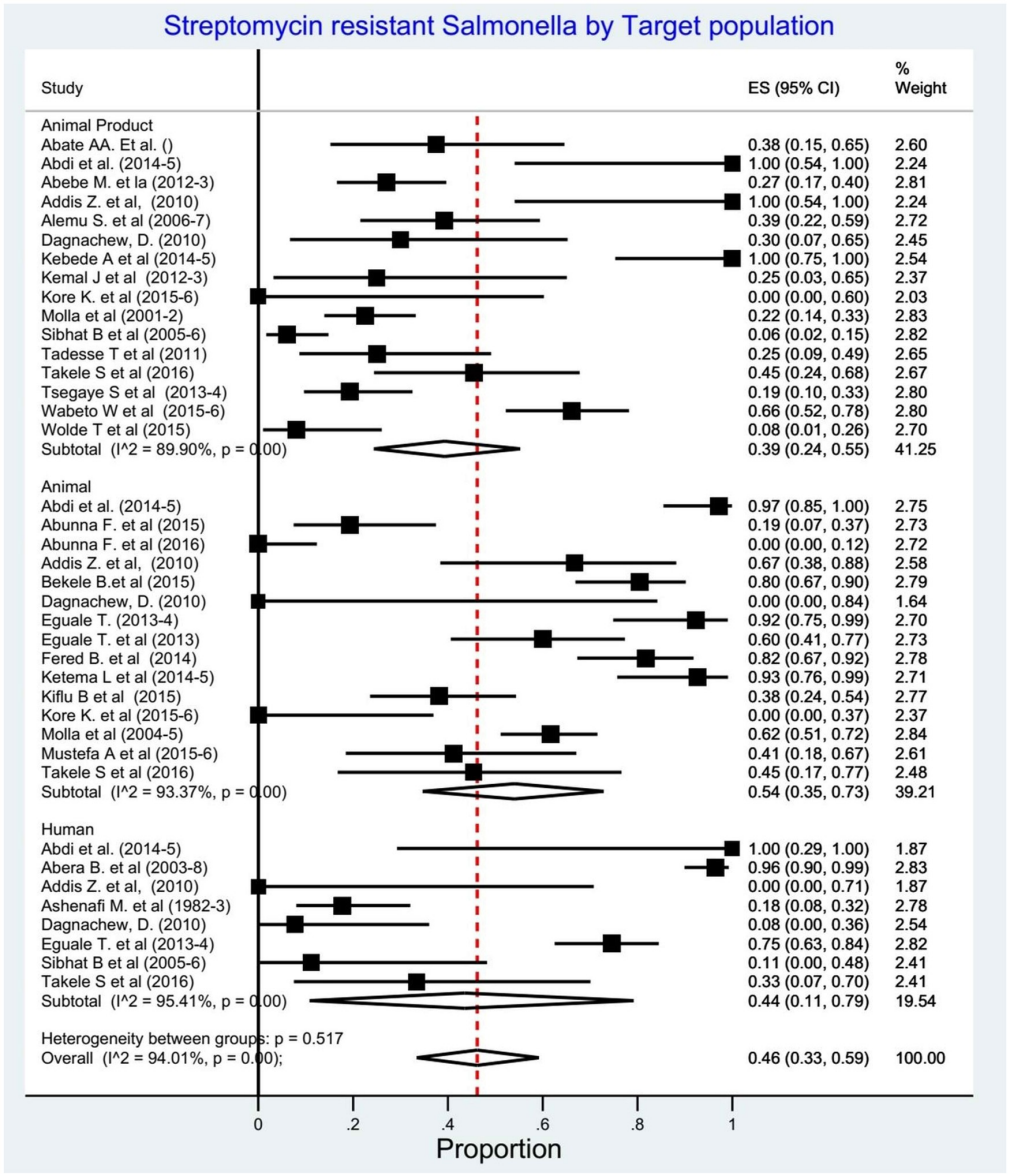
Forest plots: Proportions of streptomycin resistant *Salmonella* serovars by target population.

**Fig 9:**
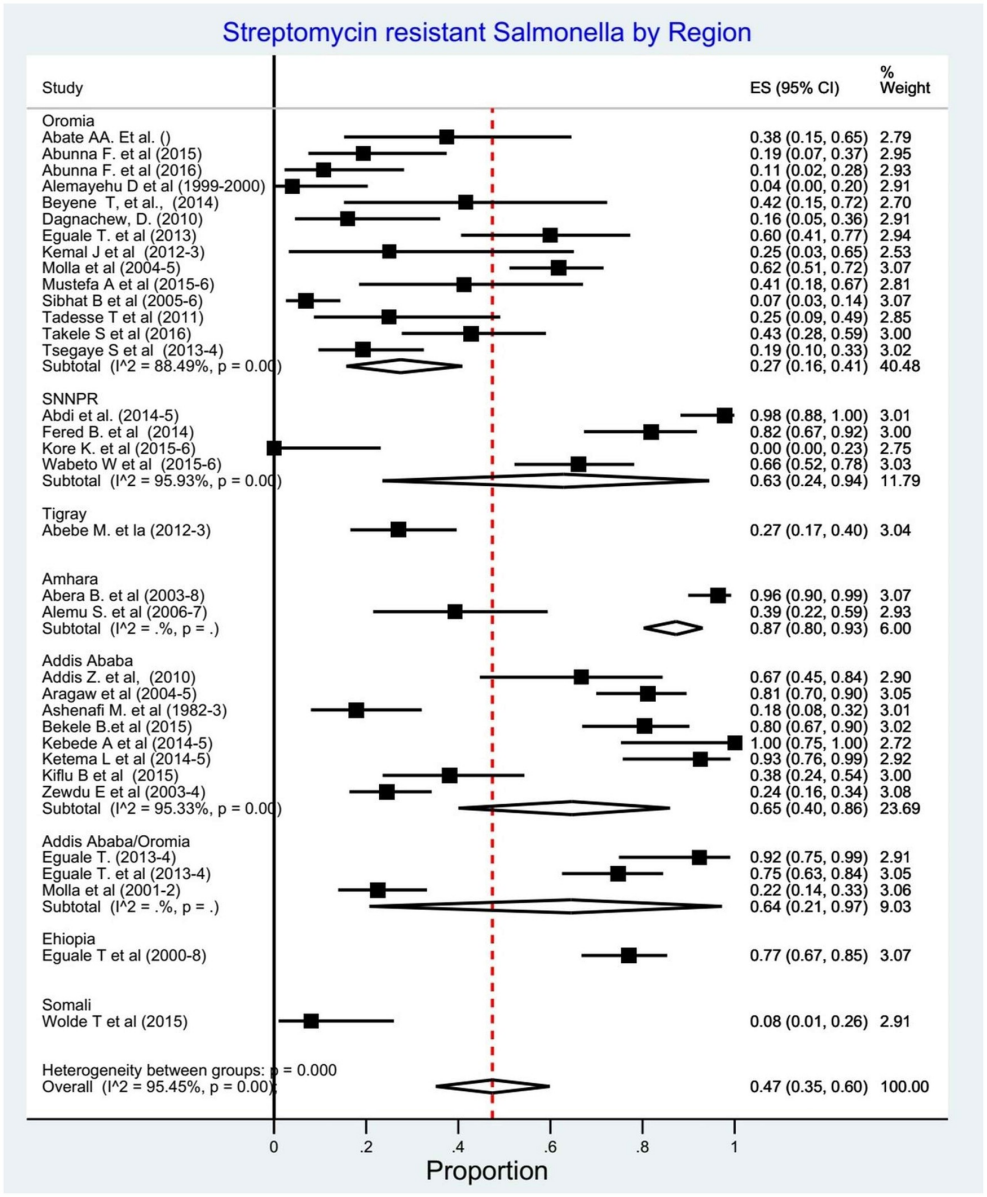
Forest plots: Proportions of streptomycin resistant *Salmonella* serovars by region.

In the subgroup analysis based on target population (n = 38), the pooled proportion of streptomycin resistant *Salmonella* serovars was highest in animals 54% (95% CI; 35–73%), followed by humans 44% (95% CI; 33–59%) and animal products 39% (95% CI;24–55%) (Fig 8). Five studies were not included in this subgroup analysis as the target population was not clearly stated [49] and the streptomycin resistance by the target population is not available [29, 33, 38, 53]. Another subgroup analysis by region, the pooled proportion of the streptomycin resistance *Salmonella serovars* was 87% (95% CI; 80–93%) in Amhara,65% (95% CI; 40–86%) in Addis Ababa and 63% (95% CI; 24–94) in SNNPR (Fig 9).

**Fig 10:**
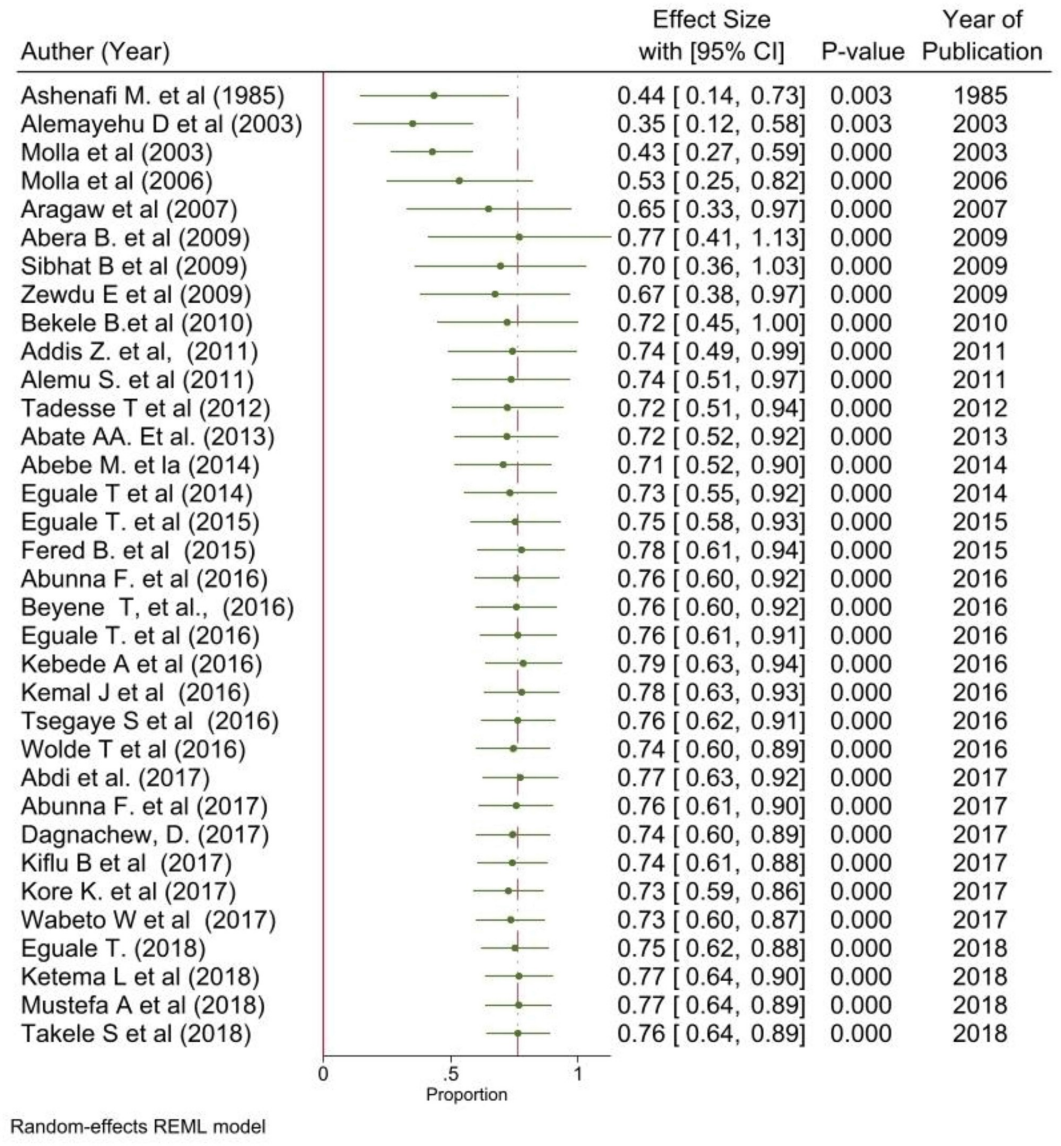
Forest plots: Cumulative analysis of proportion of streptomycin resistant *Salmonella* serovars.

The time base analysis revealed that, despite not linear, the proportion of streptomycin resistant *Salmonella* serovars increased from 0.35 (95% CI; 0.12–0.58) in 2003 to0.77 (95% CI; 0.64–0.89) in 2018 among the study population (Fig 10).

#### Sensitivity analysis

Sensitivity analysis is a vital part of meta-analysis as its goal is to determine the robustness of the observed outcomes to the assumptions considered in executing the analysis. Therefore, we did a sensitivity analysis to investigate the influence of one study on the overall meta-analysis estimate. The result presented that there is no sensitive article that influences either negatively or positivity the proportion of streptomycin resistant *Salmonella* serovars in Ethiopia.

## Discussion

Antimicrobial resistant bacteria are a worldwide problem that affects all countries globally. In the case of zoonotic disease-causing microorganisms, the prevention of emerging antibiotic resistant strains is multifaced. This means; to prevent and control AMR in such organisms, compressive data from humans, animals and animal products should be available. To the best of our knowledge, this is the first meta-analyses assessing streptomycin resistant *Salmonella* serovars among humans, animals, and foods of animal origin in Ethiopia. In the review process, we found huge number of very heterogeneous literature conducted using different study designs, varied sample sizes, and different microbiological diagnostic methods; and most of them were excluded. However, we still found an adequate number of studies that enable us to produce data to answer our research questions.

In this meta-analysis, the pooled proportion of streptomycin resistant *Salmonella* serovars among animals and humans was 54% and 44%, respectively. This is higher than a meta-analysis among poultry (22.5%) and humans (12.4%) in Brazil [55]; and Broiler Production (2.8%) in a Nigerian national study [56]. The difference might be related to the difference in *Salmonella* serovars isolated, the AMR prevention and control practice across the countries, the microbiological diagnostic methods used, or the nature of the study population. In this case, the study conducted in Nigeria was the national surveillance against the meta analysis in our cases.

A data from time series analysis of the AMR pattern is much more important than the data taken in a snap shot approach (in a single year) to evaluate the program implemented and to design alternative AMR preventive and control strategies in a country. In this study, we observed a significant increase (from 0.35 to 0.77) of the streptomycin resistant *Salmonella* serovars from 2003 to 2018. The significant level of self-antibiotic prescription, self-medication, wrong indication, wrong duration, improper route of administration, immature discontinuation of antibiotics, and use of leftover antibiotics might be the possible reasons for this change as described in the recent meta-analysis conducted in the country [57].

The emergent MDR bacteria are the worst face of the ongoing overall increase of the resistance bacterial globally. The effect is particularly worrisome in developing countries where alternative antimicrobial drugs are lacking, routine testing of the isolates for antimicrobial resistance is limited and empirical treatment is used most of the time. In our review, we found that more than half of the *Salmonella* isolates (50.1%) were MDR. This is lower than a study conducted in India (80%) [58] but higher than that seen in a continent meta-analysis conducted in Africa (34.6%) [59]. The variation could also be explained with the different drug usage policies of the countries. In addition, our finding is lower than the other study done in Ethiopia (71.4%) [60]. In their study, they determined the MDR *Salmonella* serovars for other antimicrobials in addition to streptomycin that may vary the result with this study.

This is the first systematic review and meta-analysis addressed streptomycin resistance for *Salmonella* serovars in Ethiopia. It has analyzed articles from most parts of the country where eligible studies have been retrieved from. It has included those articles that were conducted to isolate streptomycin resistant *Salmonella* serovars in Ethiopia up to the time of data extraction.

Nevertheless, this study has several limitations. The studies used for analysis were heterogeneous as some were done in healthy participants and others in patients, a different group of animals and animal products, though this was dealt with a random-effects model. It was difficult to analyze the streptomycin resistance pattern of each bacterial serovars. The studies from some regions such as Tigray and Somali are limited in number, so the study result may not be representative of the remaining part of the country. We detected the high level of heterogeneity across all analyses, so the readers should interpret the pooled analysis and subgroup with caution.

## Conclusion

In this review, about half of *Salmonella* serovars were streptomycin and multidrug resistance. This resistance was high when analyzed separately among human, animal and animal products. Moreover, a significantly increased level of streptomycin resistance was found in the last three decades. This shows a need for regular epidemiological surveillance to monitoring the occurrence and transmission of streptomycin resistance *Salmonella* serovars among human, animal and animal products. Judicial use of streptomycin in both humans and animals could prevent further development of resistance and prevention of resistant serovar transmission across the susceptible population is not left to tomorrow. Lastly, this review call to practice the one health approach to preventing further development of resistance in Ethiopia.

## Supporting information

S1 Checklist PRISMA checklist. (DOC)

S1 Supplement Characteristics of included studies. (XLSX)

S2 Supplement Methodological qualities of included studies. (XLSX)

## Abbreviations

AMR: Antimicrobial Resistance
CLSI: Clinical Laboratory Standard Institute
DST: Drug Susceptibility Test
JBI: Joanna Briggs Institute
MDR: Multi drug resistant
MIC: Minimum inhibitory concentration
MOOSE: Meta-Analysis of Observational Studies in Epidemiology
NTS: Non-typhoidal *Salmonella*
PRISMA: Preferred Reporting Items for Systematic Reviews and Meta-Analyses
SNNPR: Southern Nation Nationality and people region
USA: United States of America
ZD: Zone Diameters
